# Acute Pancreatitis Mimicking ST-Segment Elevation Myocardial Infarction

**DOI:** 10.1155/2018/9382904

**Published:** 2018-10-24

**Authors:** Akanksha Agrawal, Nuzhat Sayyida, Jorge Luis Penalver, Mary R. Ziccardi

**Affiliations:** Department of Internal Medicine, Einstein Medical Center, Philadelphia, PA, USA

## Abstract

**Introduction:**

Electrocardiographic changes imitating myocardial ischemia have been occasionally reported in patients with intra-abdominal pathology including acute pancreatitis.

**Case Report:**

A 60-year-old man with no past medical history presented to the emergency department (ED) after a syncopal episode. In ED, his vitals were stable. His ECG showed sinus bradycardia at 53 beats per minute, peaked T waves, 1 mm ST-segment elevation in leads II, III, and aVF, and 2 mm ST elevation in V3 as shown in the figures. With the concern for STEMI, he was taken for left heart catheterization (LHC) emergently, showing nonobstructive coronary artery disease (CAD). His laboratory workup was remarkable for lipase of 25,304 IU/l (normal level 8–78 IU/l). His liver function test and triglyceride level were normal. Troponin was <0.01 ng/ml. A computed tomographic exam of the abdomen revealed acute interstitial pancreatitis with a small discrete fluid collection in the uncinate process. He was treated with aggressive intravenous fluid resuscitation and was discharged on day 3.

**Discussion:**

Intra-abdominal pathologies like acute pancreatitis can lead to transient ECG changes mimicking STEMI. It is important to use ECG clues, echocardiographic findings, and clinical judgement to avoid cardiac catheterization, contrast exposure, and associated health care costs.

## 1. Introduction

Transient electrocardiographic changes imitating myocardial ischemia have been occasionally reported in patients with intra-abdominal pathology including acute pancreatitis. The occurrence of ST elevation in a patient presenting with syncope is a cause for concern and should be evaluated emergently to rule out cardiac etiology. We present a case of syncope with inferior ST-segment elevation on electrocardiogram who was diagnosed to have acute pancreatitis.

## 2. Case Report

A 60-year-old Asian male with past medical history of glaucoma presented to the emergency department (ED) after a syncopal episode. The patient was seated at his workplace when he suddenly felt moderate epigastric pain and slumped down in his chair, after which he lost consciousness. The bystanders caught him while falling to the ground from his chair. He denied a previous episode of syncope and denied having chest pain, shortness of breath, palpitations, nausea, or vomiting. He was a nonsmoker and had occasional alcohol intake.

**Figure 1:**
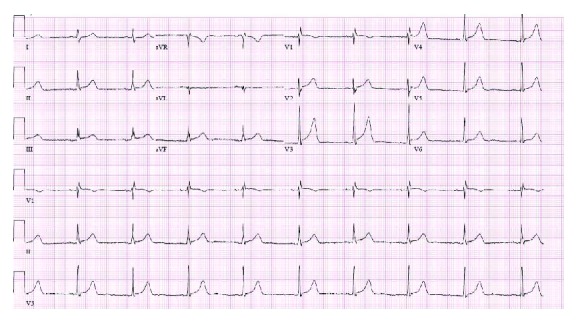
Electrocardiogram showing sinus bradycardia at 53 beats per minute, peaked T waves, 1 mm ST-segment elevation in leads II, III, and aVF, and 2 mm ST elevation in V3.

**Figure 2:**
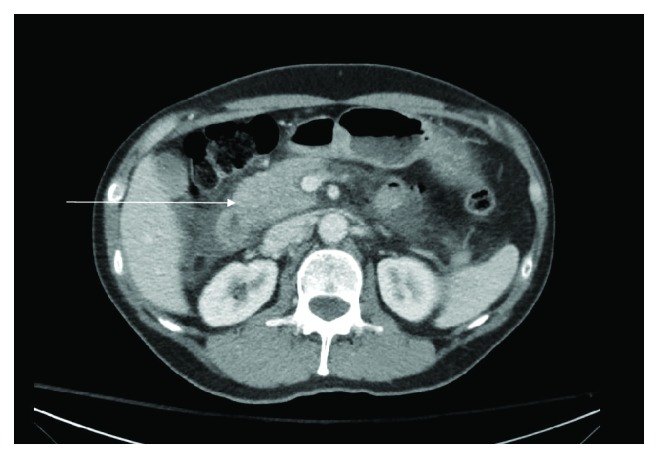
Computed tomographic image of the abdomen showing acute interstitial pancreatitis (arrow) with small discrete fluid collection in the uncinate process. Also noticeable is a moderate amount of inflammatory fluid in the anterior pararenal space and a small amount in the retroperitoneum.

In ED, his blood pressure was 125/58 mmHg, heart rate 55 beats per minute, regular, he was afebrile, and saturated 100% on room air. His electrocardiogram (ECG) showed sinus bradycardia at 53 beats per minute, peaked T waves, 1 mm ST-segment elevation in leads II, III, and aVF, and 2 mm ST elevation in V3 (Figure 1). With the concern for ST-segment elevated myocardial infarction (STEMI), he was given aspirin 324 mg and was taken for left heart catheterization (LHC) emergently. His LHC showed nonobstructive coronary artery disease (CAD). His laboratory workup was remarkable for lipase of 25,304 IU/l (normal level 8–78 IU/l) and white blood count 11,800/mcl. His liver function test, serum electrolyte level, and triglyceride level were unremarkable. Troponin was <0.01 ng/ml. A computed tomographic exam of the abdomen revealed acute interstitial pancreatitis with a small discrete fluid collection in the uncinate process (Figure 2). The ultrasound of his abdomen ruled out biliary etiology, showing a normal appearance of the gallbladder and biliary tree, without evidence of calculus or obstruction. His echocardiogram revealed normal ejection fraction with no regional wall motion abnormality.

**Figure 3:**
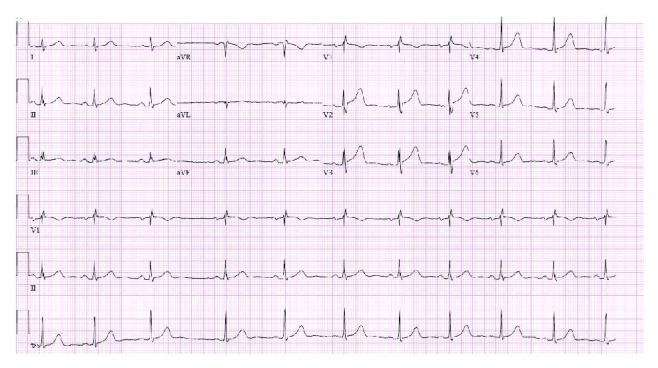
Electrocardiogram at the time of discharge of the patient showing persistent (baseline) ST-segment elevation in V2 and V3.

He was admitted to the telemetry floor and treated with aggressive intravenous fluid resuscitation. He was symptomatically better the following day and could tolerate a diet on day 3. He was discharged on day 3 with adequate follow-up. The discharging ECG is shown in Figure 3. His initial syncopal episode was thought to be a vasovagal response either due to epigastric pain or intravascular volume depletion from having severe pancreatitis.

## 3. Discussion

ST-segment elevation on ECG is commonly seen in the setting of acute coronary syndrome (ACS). However, few clinical entities simulate myocardial injury on the ECG. These include noncoronary cardiac pathologies such as pericarditis and myocarditis, vascular pathologies such as aortic dissection and pulmonary embolism, electrolyte abnormalities such as hyperkalaemia, acute intra-abdominal pathologies such as acute cholecystitis and acute pancreatitis, and miscellaneous causes including intracranial bleeds or infarction, pregnancy, limb lead reversal, hypothermia, amyloidosis, medication effects (e.g., digitalis), and illicit drug effects [1–4]. Acute pancreatitis can present like ACS with epigastric or substernal chest pain, nausea, vomiting, and syncope, making the diagnosis confusing, more so in the presence of ST-segment elevation with a concern for ischemia.

ECG abnormalities in acute pancreatitis have been described for more than 50 years. The incidence is unclear, but prior studies have shown ECG changes in around 50% of patients with acute pancreatitis [5, 6]. Rubio-Tapia et al. evaluated the ECGs of 51 patients that presented with acute pancreatitis and without preexisting heart diseases, and they found abnormal ECG findings in 55% of these patients. Within the ECG abnormalities, repolarization abnormality (20%), tachycardia (12%), and left anterior hemiblock (10%) were the most common. The most likely cause of these abnormalities was electrolyte disturbances [5]. There are few case reports describing STEMI as a complication of acute pancreatitis [7], and there are rare reports of STEMI-like ECG changes in a patient with acute pancreatitis without ACS [8, 9].

The following hypotheses have been proposed to explain these ST-segment abnormalities in ECG: (1) Hypovolemia and hypotension can cause coronary hypoperfusion especially in patients with significant coronary artery disease [10]. (2) Electrolyte abnormalities such as hypokalemia, hypomagnesemia, hypocalcemia, and hyponatremia can modify the repolarization phase on ECG [6]. (3) There is direct damage of myocytes secondary to proteolytic enzymes, such as trypsin. This can modify the permeability of the membrane, cause direct membrane injury, and induce necrosis generating an electrical disturbance and ECG abnormalities [11–13]. (4) Vasovagal stimulus secondary to the acute intra-abdominal disease can cause a cardiobiliary reflex. This vagal stimulation can cause cardiac electrical and mechanical disturbances presenting as bradycardia, AV block, hypotension, and syncope [13, 14]. (5) Other conditions, including coagulopathy, exacerbation of underlying cardiac disease, and coronary vasospasm [5], as well as stress-induced cardiomyopathy, can also be present in acute cases of pancreatitis [15, 16].

Echocardiographic evaluation of patients with chest or epigastric pain with ECG changes suggestive of acute ischemia can be helpful to determine the etiology of the symptoms. Wall motion abnormality on echocardiogram is an important finding to determine possible ischemia, though its absence does not always rule out ischemia. In patients with acute pancreatitis, transient regional wall motion abnormalities have been described in the past in the absence of coronary ischemia. These can be related to myocardial stunning. This stunning could be the result of transient coronary thrombosis or coronary vasospasm [17]. Angiographic findings in patients with pancreatitis and in patients with ST-segment elevation on ECG have shown normal coronaries in most cases [18].

Electrocardiographic changes are often noted in acute pancreatitis, though ST elevation is rarely noted. It is important to use ECG clues, echocardiographic findings, and clinical judgement to avoid cardiac catheterization, contrast exposure, and associated health care costs. Awareness of this presentation can avoid the erroneous administration of thrombolytic agents, especially in hospitals not equipped with catheterization suites, which may have fatal consequences [19]. Evaluation for noncoronary causes of ST-segment elevation might sometimes be necessary before pursuing further testing and invasive procedures.
